# Vibration analysis of a high-pressure multistage centrifugal pump

**DOI:** 10.1038/s41598-022-22605-2

**Published:** 2022-11-24

**Authors:** Yan Zhang, Jingting Liu, Xinzhen Yang, Hongmin Li, Songying Chen, Wei Lv, Wenchao Xu, Jianping Zheng, Dianyuan Wang

**Affiliations:** 1 Key Laboratory of High-Efficiency and Clean Mechanical Manufacture, School of Mechanical Engineering, Shandong University, Jinan, 250061 People’s Republic of China; 2 Binzhou Special Equipment Inspection & Research Institute, Binzhou, 256600 People’s Republic of China; 3 YanTai LongGang Pump Industry CO., LTD, Yantai, 264003 People’s Republic of China

**Keywords:** Mechanical engineering, Applied physics, Fluid dynamics

## Abstract

High-pressure multistage centrifugal pumps have been widely used in modern industry and required low vibration and noise. In this study, modal analysis of the rotor system of a seven-stage centrifugal pump was carried out numerically by introducing fluid force to ensure that the centrifugal pump would not resonate. A vibration test bench was established to investigate the characteristics with flow rates of 0.8Q_d_, 1.0Q_d,_ and 1.2Q_d_, and the vibration data of ten measuring points were collected. The period of the vibration at the bearing was found to be around 20 ms and the period was related to the shaft frequency (SF) and the blade passing frequency (BPF). The vibration of the pump casing was mainly determined by the SF, two times the SF, and two times the BPF. Mechanical motion is the main factor causing pump vibration, and fluid unstable motion is also an important cause.

## Introduction

Multistage centrifugal pumps are an important piece of equipment for fluid transportation based on the single-stage centrifugal pump, which could provide high-pressure liquid and is widely used in agriculture and industry^1–3^. The modern industry has put forward higher requirements for the vibration of multi-stage centrifugal pumps^4–6^. The vibration problem of centrifugal pumps is bound to bring challenges to the safety and stability of operation^7,8^. Vibration analysis plays an important role in the condition detection and fault diagnosis of multistage centrifugal pumps^9,10^. The vibration problem of multistage centrifugal pumps is mainly reflected in the rotor system. When the rotor speed is close to the critical speed, it may even cause resonance and cause huge harm^11,12^. Under the premise of avoiding the resonance of the rotor system, it is of great significance to study the vibration characteristics of multistage centrifugal pumps to ensure their safe operation. However, the current research in this area mostly focuses on single-stage centrifugal pumps, and there are few reports of multistage centrifugal pumps.

Modal analysis can predict the resonance condition of centrifugal pumps by extracting mode shapes, natural frequencies, and critical speeds, which is an effective method to analyze the vibration characteristics of centrifugal pumps^13^. Sendilvelan et al.^14^ conducted modal analysis on centrifugal pump impellers with different thicknesses and extracted the natural frequencies and mode shapes of the impeller. He et al.^15^ analyzed the natural vibration and critical speeds of the rotor of a multistage centrifugal pump with different support stiffnesses and found that the first and second critical speeds were greatly affected by the support stiffness. Tian et al.^16^ found that support stiffness and fluid action had an important impact on the critical speed of the multistage centrifugal pump rotor. Ashri et al.^17^ studied the natural frequencies and mode shapes of a centrifugal pump impeller with the finite element method and found that the impeller thickness had a great influence on the natural frequency. Zhao et al.^18^ studied the resonance characteristics of a large centrifugal pump rotor system by calculating the natural frequencies and critical speeds by the finite element method. Ping^19^ studied the effect of the interstage sealing gap of a centrifugal pump on the critical speeds by combining numerical simulation and experiment. Many factors can affect the natural frequency and critical speed of a centrifugal pump rotor. However, the fluid force and the constraints of the rotor system must be considered.

To adapt to the increasingly high operating requirements of centrifugal pumps, many scholars have studied the vibration characteristics of centrifugal pumps. Kato et al.^20^ analyzed the vibration of a multi-stage centrifugal pump by one-way fluid–structure interaction and found that the vibration mainly originated from the interaction between the rotor and the stator. Dai et al.^21^ studied the effect of fluid excitation on a marine centrifugal pump and found that the dominant frequency of vibration was the blade passing frequency. Jiang et al.^22^ studied the vibration and noise of a five-stage centrifugal pump using the fluid–structure coupling method. Chen et al.^23^ modeled the vibration and noise caused by a centrifugal pump and found that the dominant frequency of the volute vibration velocity was the blade passing frequency. Rao^24^ found that the pressure at the volute tongue of centrifugal pumps was greatly affected by the blade passing frequency. Guo^25^ used the fluid–structure interaction method to analyze the rotor vibration characteristics of a centrifugal pump, and the pressure pulsation showed periodic changes. The work of these scholars provided an experience for studying the frequency characteristics of centrifugal pumps, but there are also problems in the research process that there are few measuring points, and the analysis of the frequency characteristics of different positions is not comprehensive.

In actual operation, centrifugal pumps may not work at the rated flow, so it is necessary to study the vibration characteristics of centrifugal pumps at different flow rates. Behzad et al.^26^ conducted vibration tests of a centrifugal pump at different flow rates and found that operation under non-design conditions was one of the reasons for the aggravated vibration. Khalifa^27^ studied the vibration characteristics of a single-stage double-volute centrifugal pump at different flow rates and found that the vibration increased at non-rated conditions. Al-Obaidi et al.^28^ studied the influence of different flow rates on the performance and cavitation of a centrifugal pump based on vibration analysis techniques. Bai et al.^29^ studied the vibration and stability of a multistage centrifugal pump at different flow rates and found that the blade passing frequency and 2 times the blade passing frequency were the main excitation frequencies. Lu et al.^30^ measured the vibration spectrum of a centrifugal pump at three flow rates and found that the vibration velocity was the smallest at the rated flow rate. Although some scholars studied the vibration characteristics of centrifugal pumps at different flow rates, there are few studies on multi-stage centrifugal pumps with more complex structures.

In the present study, a seven-stage double-shell high-pressure centrifugal pump was taken as the research object. The critical speeds and mode shapes of the centrifugal pump rotor were analyzed by using the fluid–structure interaction method to introduce fluid force. The vibration responses of ten measuring points were obtained by the vibration test at different flow rates, and the test results were analyzed to reveal the vibration characteristics of the multistage centrifugal pump. All the results could enrich the existing database and provide a basis for further research on the properties of multistage centrifugal pumps.

## Fluid–structure interaction simulation

### Model and meshing

**Figure 1:**
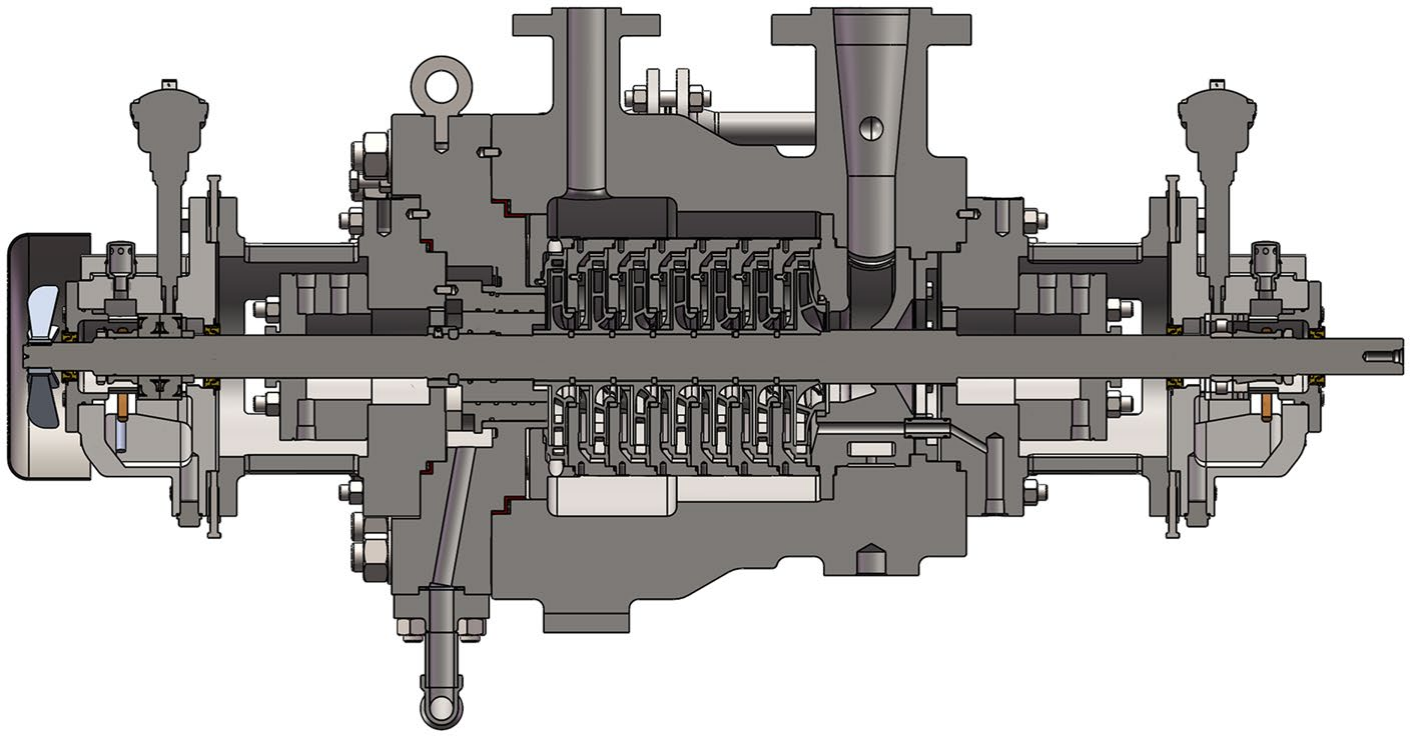
Inside of the 7-stage double-shell high-pressure water injection centrifugal pump.

The relevant parameters of the centrifugal pump are shown in Table 1, where D_in_ is the diameter of the inlet pipe and D_out_ is the diameter of the outlet pipe. The impeller parameter values are shown in Table 2. The increase in the number of blades could improve the head of centrifugal pumps, but it also increased the friction loss of the liquid, which was prone to cavitation. To improve the anti-cavitation performance of the centrifugal pump, the first-stage impeller was designed with four blades, and the remaining six-stage impellers were designed with five blades. The difference between the remaining six-stage impellers was only the angle of installation on the pump shaft. The overall cross-sectional view of the multistage centrifugal pump is shown in Fig. 1.

**Table 1:** Parameters of the multistage centrifugal pump.

Type	Q_d_ (m^3^/h)	n (rpm)	H (m)	D_in_ (mm)	D_out_ (mm)
BB5	30	2,980	245	80	40

**Table 2:** Structural parameters of the impellers.

	Inlet diameter (mm)	Inlet width (mm)	Outlet diameter (mm)	Outlet width (mm)	Hub diameter (mm)	Outlet angle	Number of blades
First impeller	94.6	12	185	11.8	56	20°	4
Other impellers	82.6	10	185	11.8	60	23°	5

**Figure 2:**
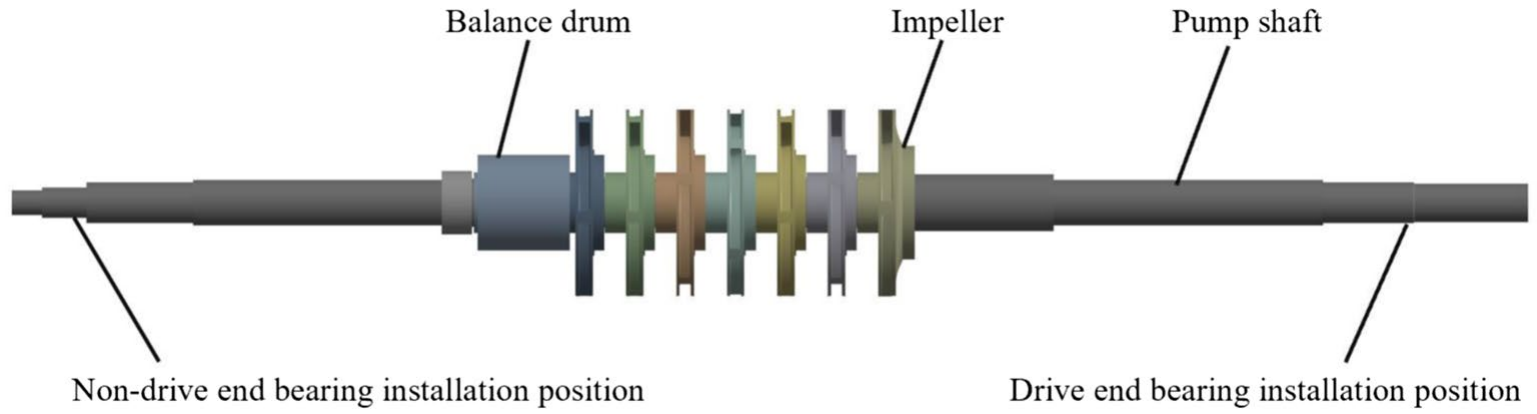
Rotor model of the centrifugal pump.

As shown in Fig. 2, the rotor system mainly included the pump shaft, the impellers, and the balance drum. The material of the pump shaft, the impellers, and the balance drum were 42CrMo, ZG1Cr13NiMo, and 30Cr13 respectively. The length of the pump shaft was 1503 mm. There were bearings on both sides of the pump shaft with a spacing of 1293 mm. A cylindrical roller bearing was installed on the drive end to withstand radial force, and an angular contact ball bearing was installed on the non-drive end to withstand radial and axial forces.

**Figure 3:**
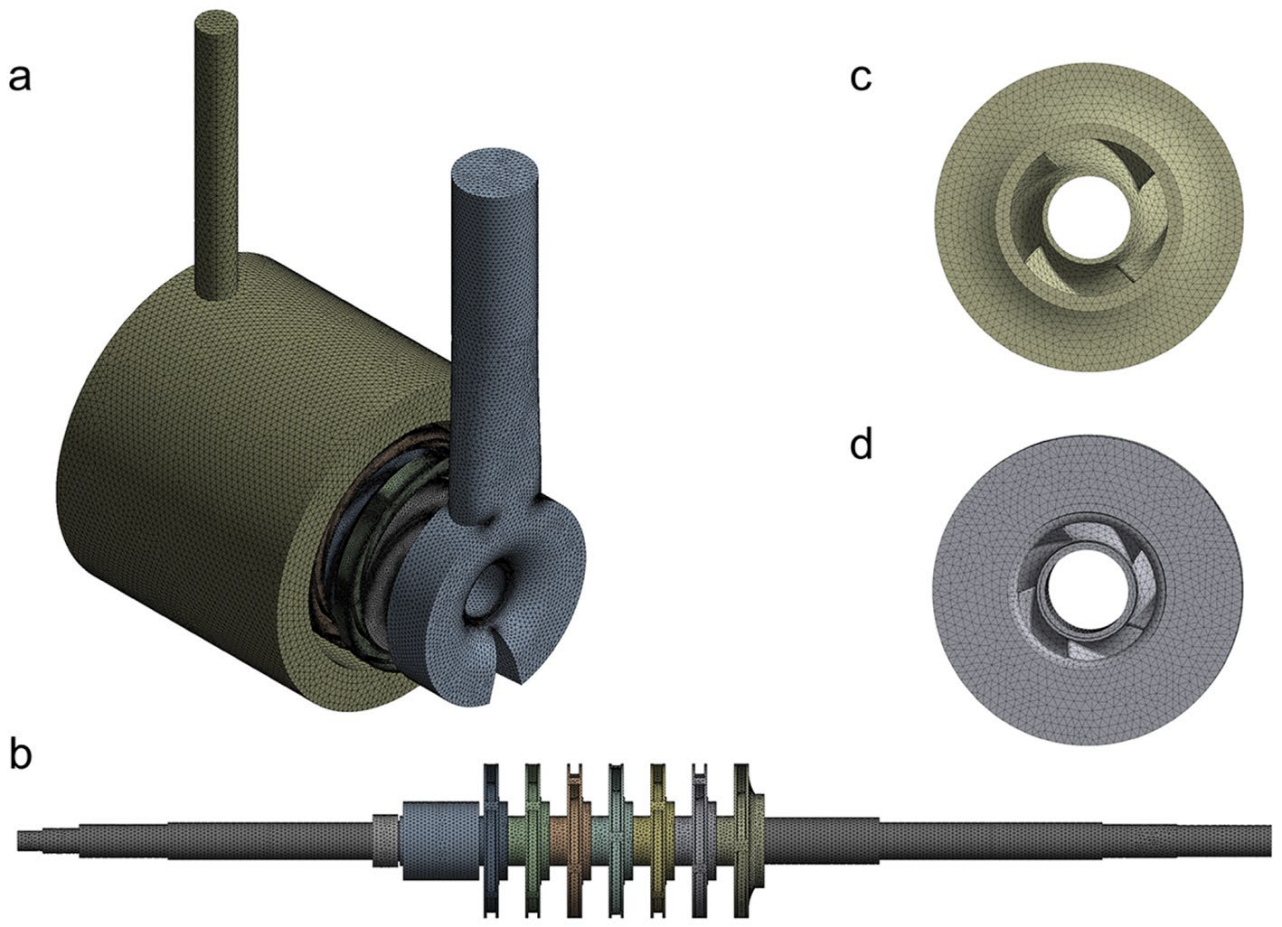
Fluid domain and solid domain meshing (**a**) Fluid domain. (**b**) Solid domain. (**c**) First impeller. (**d**) Other impellers.

Considering the complexity of the multistage centrifugal pump geometry, the unstructured tetrahedral mesh was used to partition the fluid domain and rotor system. The meshing of the model's computational domain is shown in Fig. 3. The total number of the fluid domain cells was about 9.83 million, and the average quality was 0.83. The total number of solid domain cells was 414,691, and the average quality was 0.75.

### Fluid–structure interaction

Fluid–structure interaction was a method to study the interaction between fluid and solid domains. According to the mechanism of action, it could be divided into two coupling effects. One was the strong interaction of the two-way action between the fluid domain and the solid domain, and the other was the weak interaction of the one-way action of the fluid domain to the solid domain. Since the deformation of the rotor system in this study was very small, the effect of the solid domain on the fluid domain could be ignored, so a weak interaction strategy was used for analysis.

ANSYS Fluent 18.2 was used to model the fluid domain of the centrifugal pump. The fluid was water, with a temperature of 20 °C, and a density of 998.2 kg/m^3^. A rotating reference frame was adopted to set the impeller fluid domain as a rotating fluid domain at 2980 rpm, and the remaining fluid domains as stationary regions. Each wall of the impeller fluid domain was set as a rotating wall at 2980 rpm, while the other fluid domain walls were set as stationary walls. The inlet boundary was set as the pressure inlet, and the inlet pressure was set to − 9400 Pa according to the actual pressure measured by the centrifugal pump at the rated flow. The outlet boundary was set as the mass flow outlet. The turbulence model was set as the RNG *k–ε* model. The no-slip boundary condition was used and the standard wall function was selected for the treatment of the near wall. The pressure basis-based solver was selected and the SIMPLE algorithm was used. Gravitational acceleration was set to − 9.81 m/s^2^. Convergence residuals were set to 10e−4 to ensure computational convergence. The error between the head (255.5 m) obtained by the fluid domain simulation and the actual head (245 m) was about 4.3%. The fluid domain simulation results were relatively accurate.

**Figure 4:**
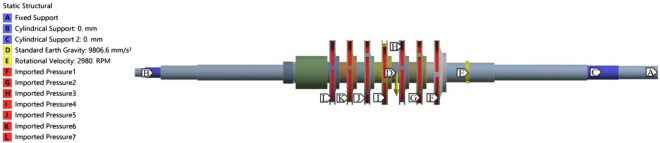
Loads and restraints for the centrifugal pump rotor.

The solid domain was solved in ANSYS Workbench 18.2. In addition to centrifugal force and gravity, the rotor system was also subjected to fluid forces which included the pressure load on the impeller blades and hub. The centrifugal force was applied by setting the rotational speed to 2980 rpm. The gravity was applied by setting the gravitational acceleration to − 9.81 m/s^2^. The fluid forces were introduced by importing the fluid domain calculation at the fluid–structure interaction surface (the flow surface in the impeller). The cylindrical constraint method was used to constrain the contact points between the shaft and the bearing at both ends, and the axial and radial directions were restricted while the tangential direction was kept free. Since the shaft end near the motor was connected to the motor by a coupling, the fixed constraint method was used to constrain the shaft end. The force and constraint of the rotor system under the condition of fluid–structure interaction are shown in Fig. 4.

## Modal analysis

**Figure 5:**
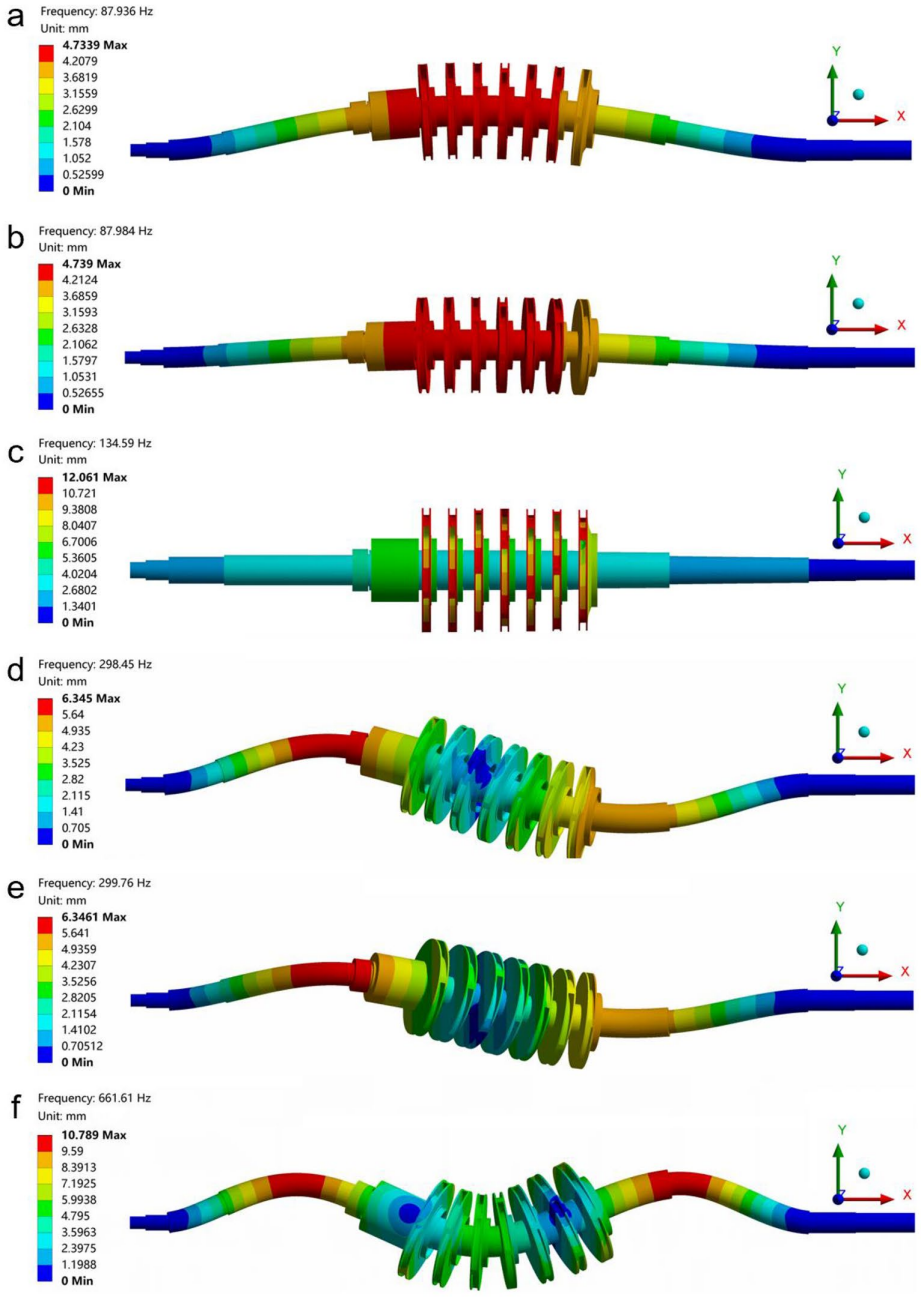
The first six mode shapes of the centrifugal pump rotor system. (**a**) First mode shape. (**b**) Second mode shape. (**c**) Third mode shape. (**d**) Fourth mode shape. (**e**) Fifth mode shape. (**f**) Sixth mode shape.

After applying loads and constraints, the model was solved in ANSYS Workbench 18.2. Modal analysis was a method for calculating the dynamic characteristics of linear structures. The vibration characteristics of linear structures in the sensitive frequency range could be calculated and the vibration response could be predicted by modal analysis. Since it was usually the lower eigenfrequency that caused the structural resonance of the rotor system, the first six modes were extracted by the Block Lancos method to obtain the natural frequencies and the mode shapes of the rotor system. The first six natural frequencies of the rotor system are shown in Table 3, and the mode shapes are shown in Fig. 5.

**Table 3:** The first six natural frequencies of the rotor system (unit: Hz).

Mode	1	2	3	4	5	6
Natural frequency (Hz)	87.936	87.984	134.59	298.45	299.76	661.61

The mode shapes of the rotor system mainly included swing vibration, torsional vibration, bending vibration, and pitching vibration. The first natural frequency was 87.948 Hz. The mode shape showed up and down swings along the Y and Z directions. The second natural frequency was 88 Hz. The mode shape showed up and down swings along the Z and Y directions, which was orthogonal to the first mode shape. The third natural frequency was 135.31 Hz. The mode shape showed torsional vibration in the YZ plane and up and down swings in the Y and Z directions. The fourth natural frequency was 298.34 Hz. The mode shape showed the S-shaped bending vibration in the XY plane. The fifth natural frequency was 299.66 Hz. The mode shape exhibited S-shaped bending vibration in the XZ plane, which was orthogonal to the fourth mode shape. The sixth natural frequency was 661.42 Hz. The mode shape showed the pitch vibration along the X direction.

**Figure 6:**
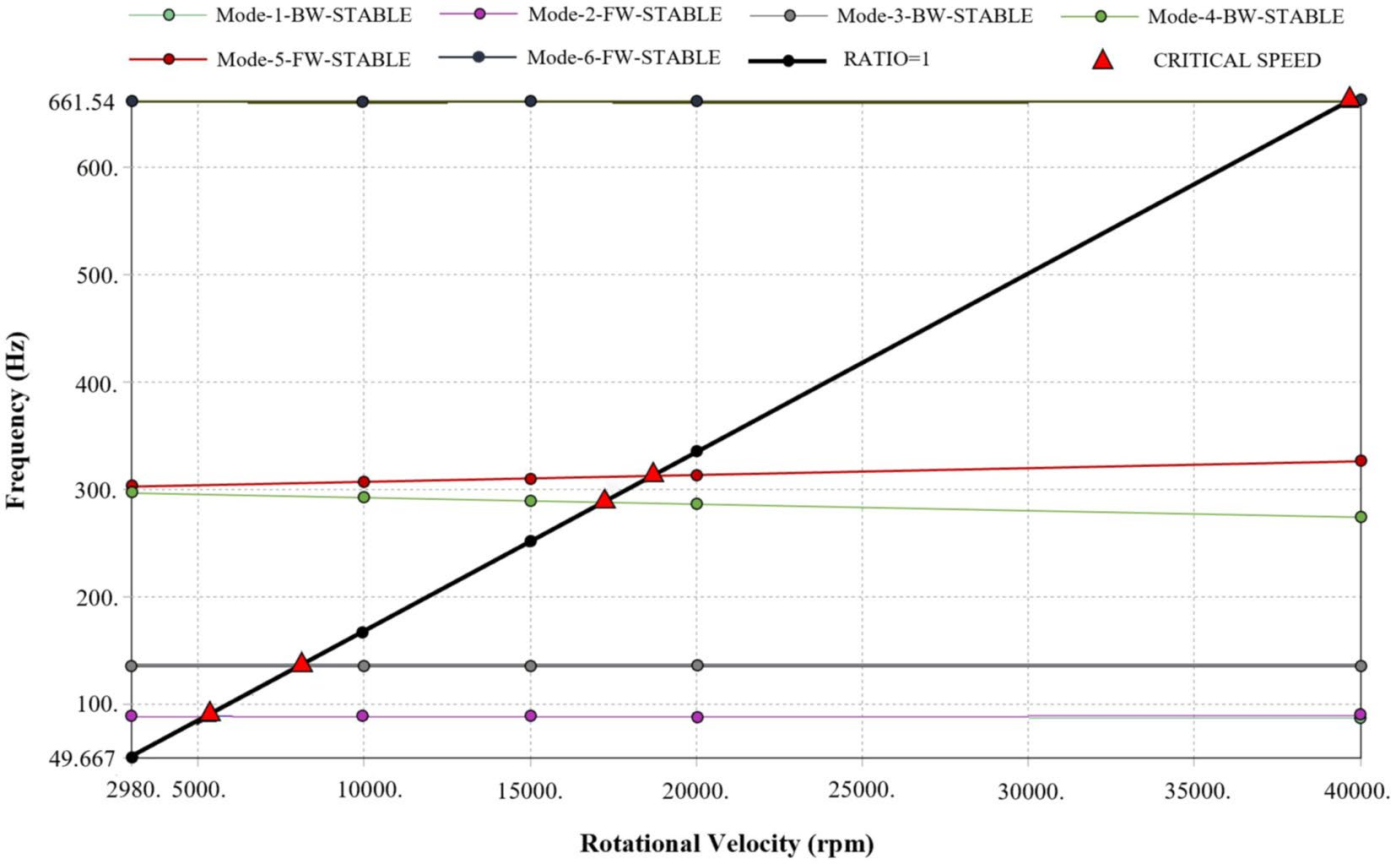
Campbell diagram.

The Campbell diagram is shown in Fig. 6. The oblique line from the origin represented the frequency of the exciting force, and the remaining lines represented the natural frequency. When the oblique line and the natural frequency line intersect, it indicated that resonance may occur. It could be seen from Fig. 6 that the first critical speed of the rotor system was 5270.9 rpm, which was much higher than the rated speed of 2980 rpm. Therefore, the rotor system of the centrifugal pump would not resonate. The critical speeds of the first six modes are shown in Table 4.

**Table 4:** Critical speeds of the rotor system.

Mode	1	2	3	4	5	6
Critical speed (rpm)	5270.9	5286	8118.8	17,274	18,690	39,685

## Vibration tests of the centrifugal pump

### Test device

**Figure 7:**
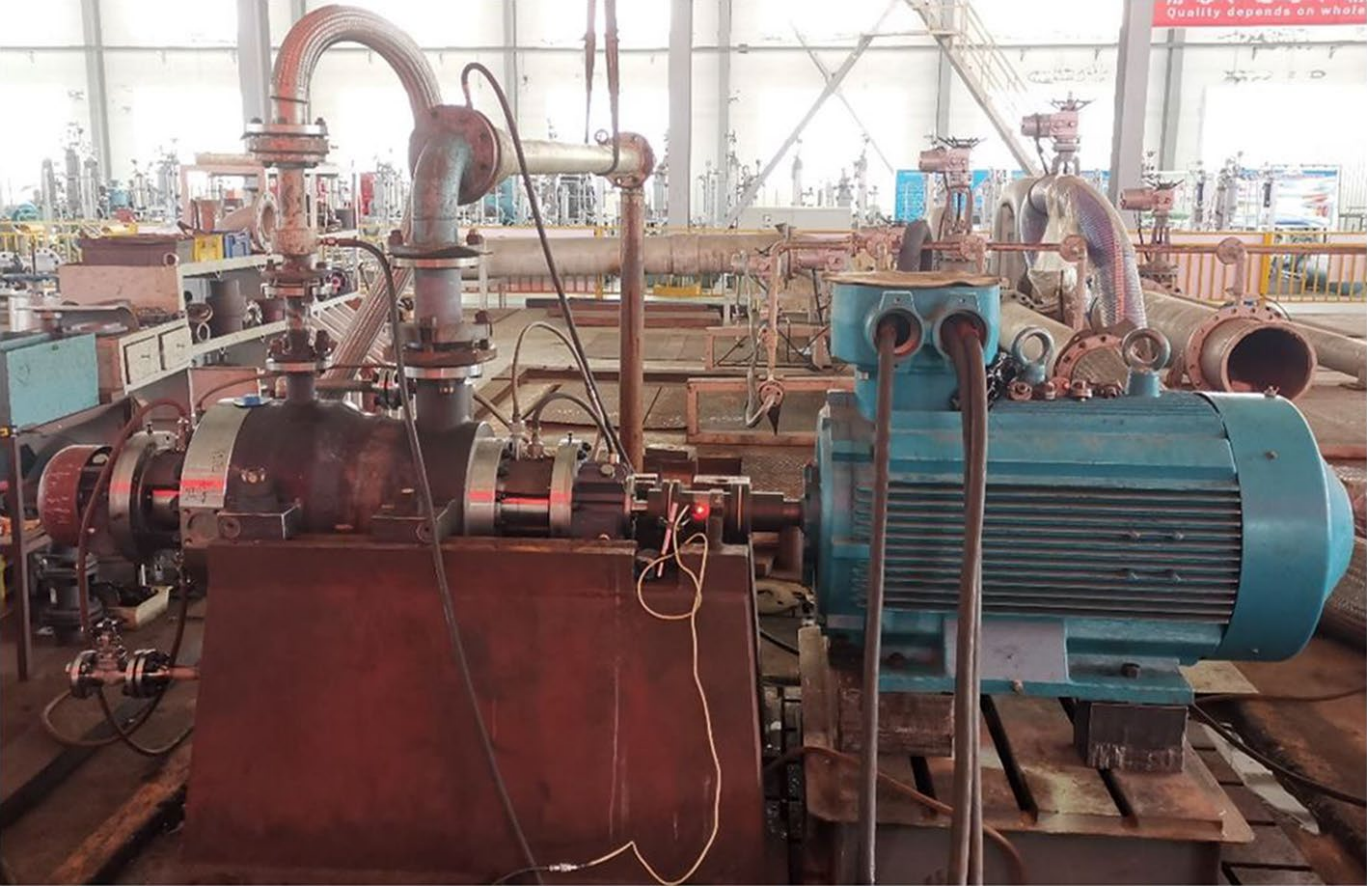
Centrifugal pump test platform.

**Figure 8:**
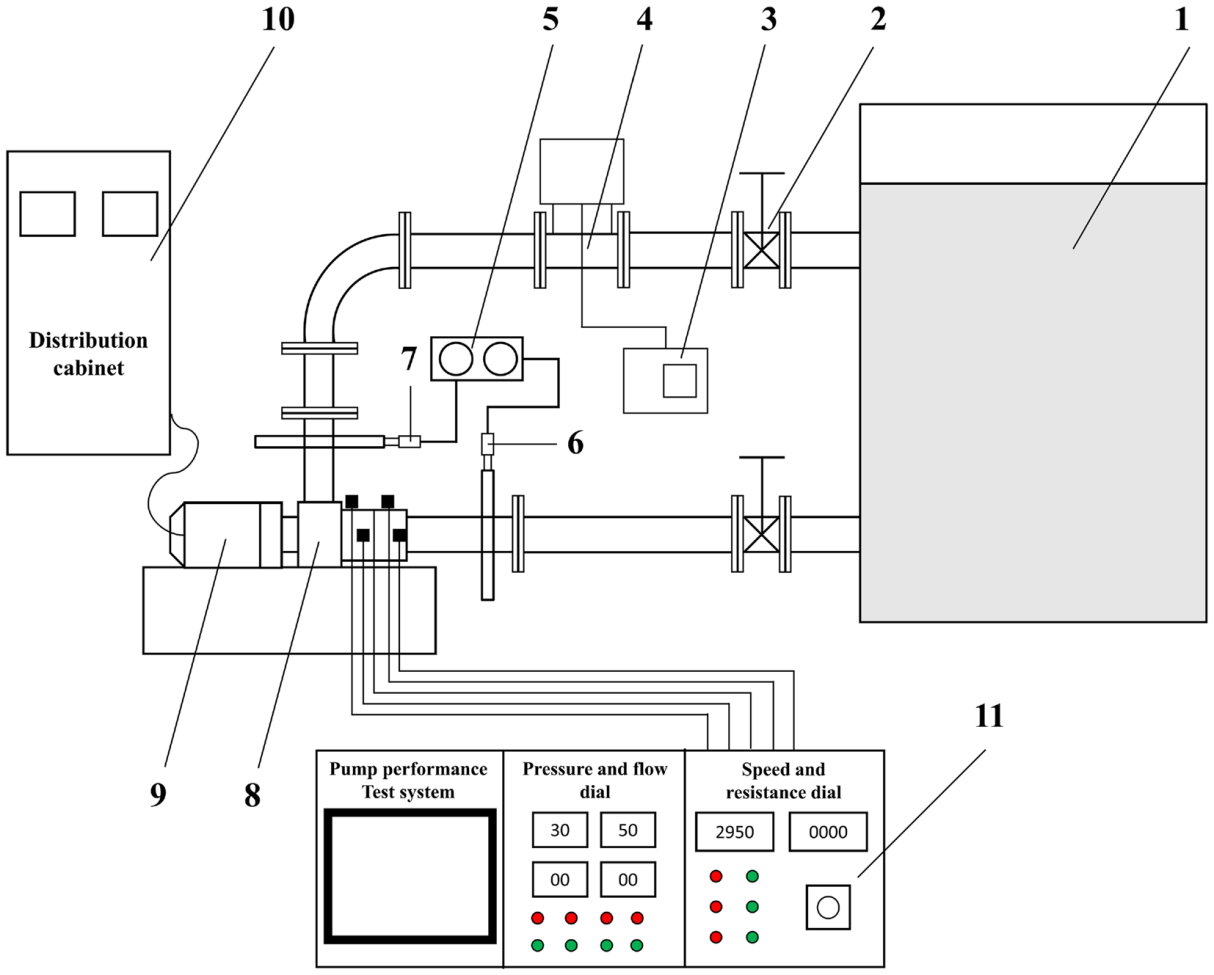
Schematic diagram of the centrifugal pump test device. (1) Water tank, (2) Control valve, (3) Flowmeter indicator, (4) Electromagnetic flowmeter, (5) Pressure indicator, (6) Pressure sensor (inlet), (7) Pressure sensor (outlet), (8) 7-stage centrifugal pump, (9) Motor, (10) Distribution cabinet, (11) Console.

The centrifugal pump test platform mainly included the 7-stage double-shell high-pressure water injection centrifugal pump, water tank, inlet pipeline, outlet pipeline, motor, pump performance test system, digital collector, pressure gauge, electromagnetic flowmeter, regulating valve, etc. After testing, the installation of the test platform was firm, which could ensure the smooth operation of the multi-stage centrifugal pump. The centrifugal pump test platform is shown in Fig. 7 and the schematic diagram of the test device is shown in Fig. 8. The content of the experimental research was to measure the vibration of the seven-stage double-shell high-pressure centrifugal pump. In this experiment, the ZX601A data collector and L14A piezoelectric accelerometer were used to measure the vibration velocity of the centrifugal pump. The parameters of L14A are shown in Table 5.

**Table 5:** Parameters of the L14A sensor.

Name	Type	Made	Range (Hz)	Sensitivity (pC/ms^−2^)	Error
L14A	Piezoelectric accelerometer	SENDIG	2–3000	5.51	5%

### Test positions

**Figure 9:**
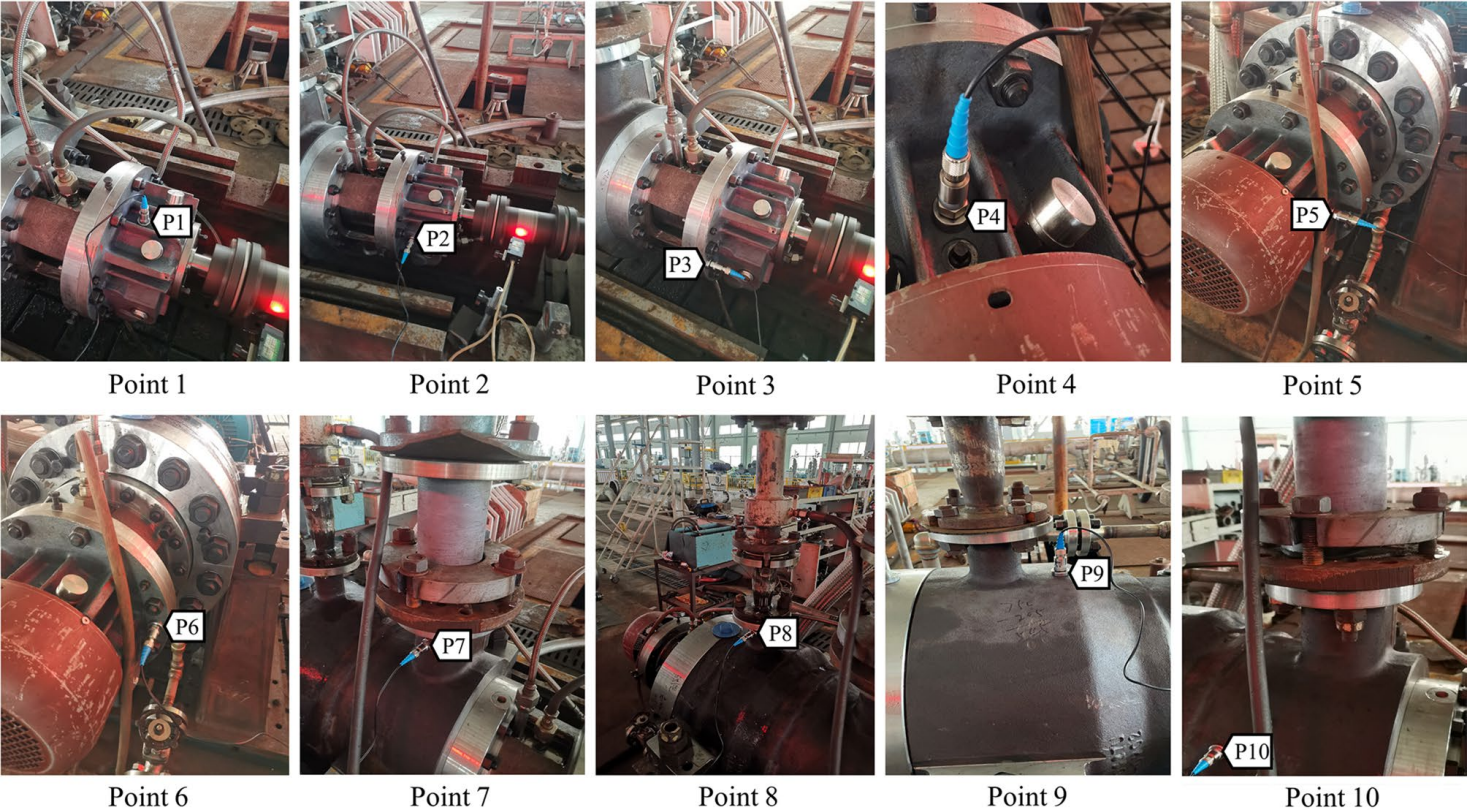
Arrangement of measuring points.

The vertical, horizontal, and axial vibration directions of the drive end and non-drive end bearings were measured to reflect the vibration of the centrifugal pump rotor system. In addition, the vertical and horizontal directions of the pump casing and the horizontal direction of the inlet and outlet pipes were selected to reflect the overall vibration of the centrifugal pump. The position of each measuring point is shown in Table 6. The layout of the measuring points is shown in Fig. 9. The flow rate was adjusted to 0.8Qd (24 m^3^/h), 1.0Qd (30 m^3^/h), and 1.2Qd (36 m^3^/h) by controlling the valve opening, and vibration tests were performed on each measuring point.

**Table 6:** Position of 10 measuring points.

Number	Measuring point	Position
1	Point 1	The vertical direction of the drive end bearing
2	Point 2	The horizontal direction of the drive end bearing
3	Point 3	The axial direction of the drive end bearing
4	Point 4	The vertical direction of the non-drive end bearing
5	Point 5	The horizontal direction of the non-drive end bearing
6	Point 6	The axial direction of the non-drive end bearing
7	Point 7	The vertical direction of the inlet pipe
8	Point 8	The vertical direction of the outlet pipe
9	Point 9	The vertical direction of the pump shell
10	Point 10	The horizontal direction of the pump shell

### Vibration characteristic test results and analysis

#### Vibration analysis at rated flow

**Figure 10:**
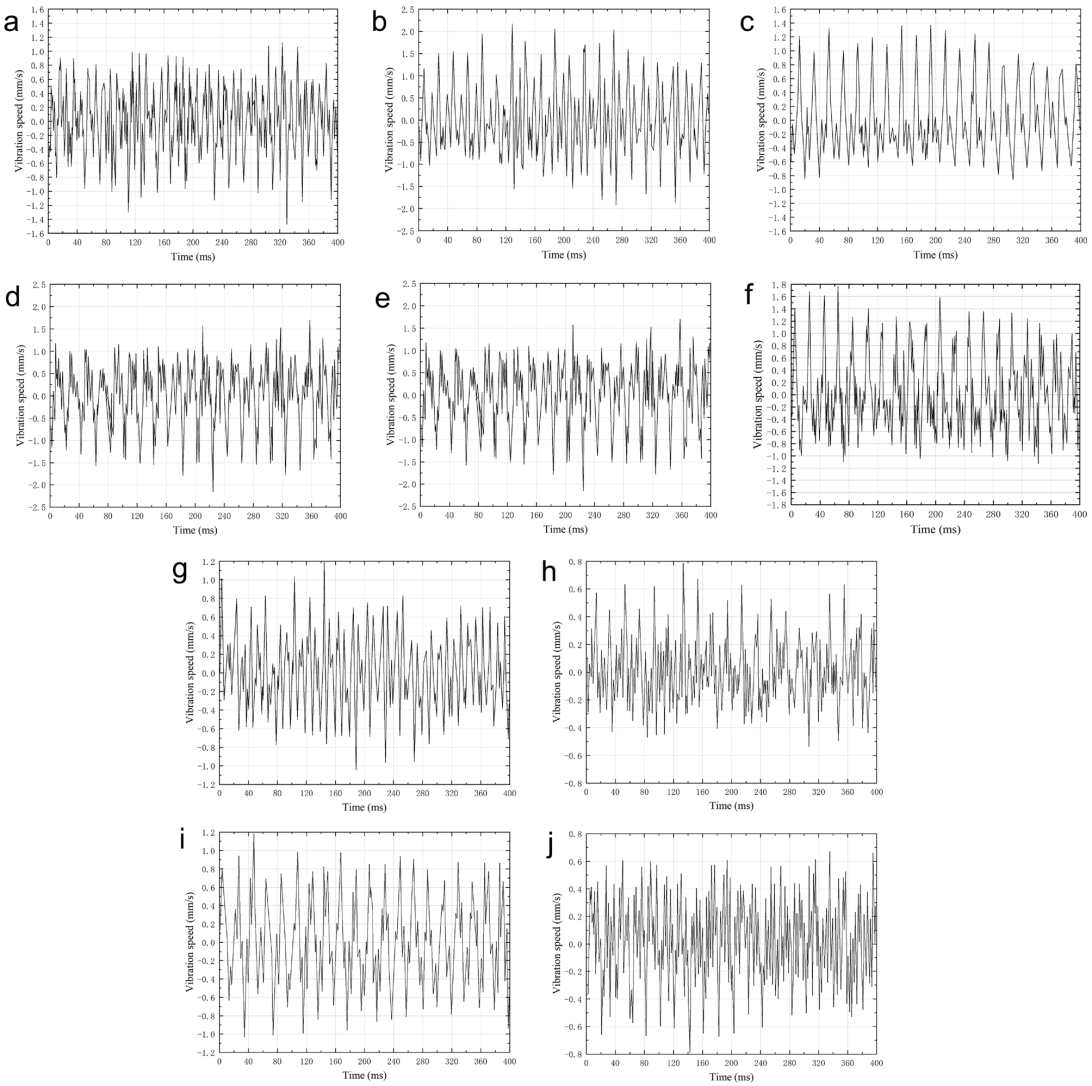
Time-domain waveforms of vibration speed at rated flow. (**a**) Point 1. (**b**) Point 2. (**c**) Point 3. (**d**) Point 4. (**e**) Point 5. (**f**) Point 6. (**g**) Point 7. (**h**) Point 8. (**i**) Point 9. (**j**) Point 10.

The time-domain waveforms of the vibration velocity of ten vibration measuring points at rated flow are shown in Fig. 10. It took 20.13 ms for the impellers to rotate once at the rated speed (2980 rpm). Observed the time-domain waveforms and found that there were about twenty regular large wave crests within the time of the impellers rotating for 400 ms. There were four to five small wave crests in the vibration period of a single bearing, which corresponded to the number of the impeller blades. This proved that the vibration law was not only related to the shaft frequency but also closely related to the blade passing frequency.

**Figure 11:**
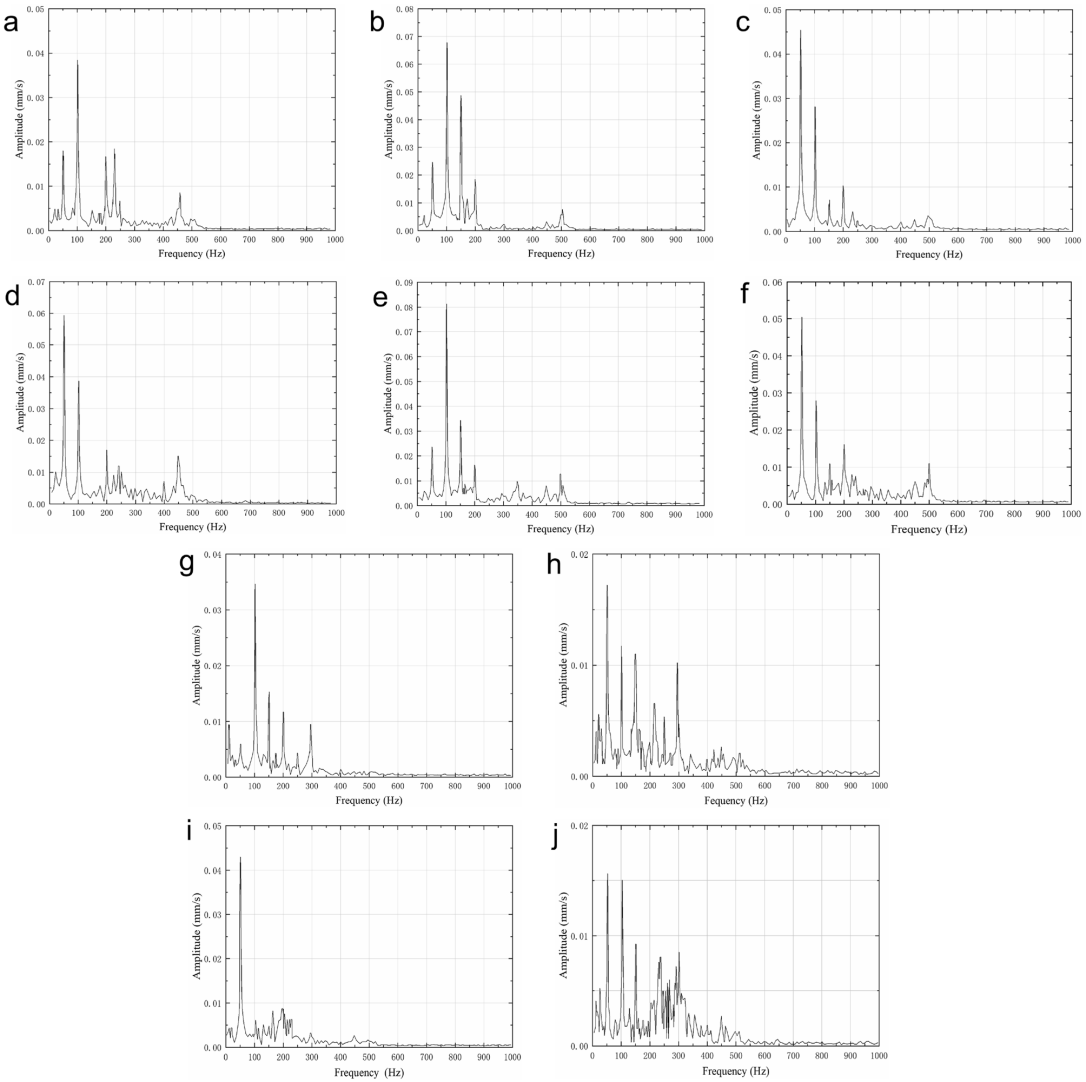
Frequency-domain waveforms of vibration speed at rated flow. (**a**) Point 1. (**b**) Point 2. (**c**) Point 3. (**d**) Point 4. (**e**) Point 5. (**f**) Point 6. (**g**) Point 7. (**h**) Point 8. (**i**) Point 9. (**j**) Point 10.

The frequency-domain waveforms of the vibration velocity of ten vibration measuring points at rated flow are shown in Fig. 11. Observing the ten measuring points, it could be found that the vibration velocity of the three measuring points at the non-drive end bearing was generally higher than that of the three measuring points at the drive end bearing. The reason was that the pump shaft and the motor were connected at the drive end bearing by a coupling, and the drive bearing end reduced its vibration intensity due to the fixed support. The vibration speed of the four measuring points corresponding to the inlet and outlet pipes and the pump casing was smaller than that of the bearing, indicating that the pump was well fixed and the flow field was stable.

The shaft frequency *f*_pf_ of the centrifugal pump could be calculated by the rotation speed (*f*_pf_ = n/60 ≈ 49.7 Hz), and the blade passing frequency *f*_bpf_ could be obtained by the number *z* of the impeller blades (*f*_bpf_ = z*f_pf_ ≈198.6–248.3 Hz). The frequency-domain waveforms showed that the vibration peaks of the multistage centrifugal pump all appeared at the shaft frequency and its frequency multiplication. The maximum peaks appeared at the shaft frequency and 2 times the shaft frequency. In addition, certain peaks also appeared in the frequency range of 200–250 Hz and 400–500 Hz, which were closely related to the structure of the multistage centrifugal pump itself. The number of blades of the first-stage impeller, the remaining six impellers, and the diffusers was four, five, and six respectively. Table 7 shows the expected blade passing frequency of multi-stage centrifugal pumps. The expected blade passing frequency was four-five times the shaft frequency, which was consistent with the results of the frequency-domain waveforms.

**Table 7:** Expected centrifugal pump blade-passing frequency.

Number of diffuser blades	Number of impeller blades						
	3	4	5	6	7	8	9
4	3	–	5	3	7	2	9
5	6	4	–	6	14	16	9
6	–	4	5	–	7	4	3
7	6	8	15	6	–	8	27
8	9	–	15	9	7	–	9

#### Vibration analysis at different flow rates

**Figure 12:**
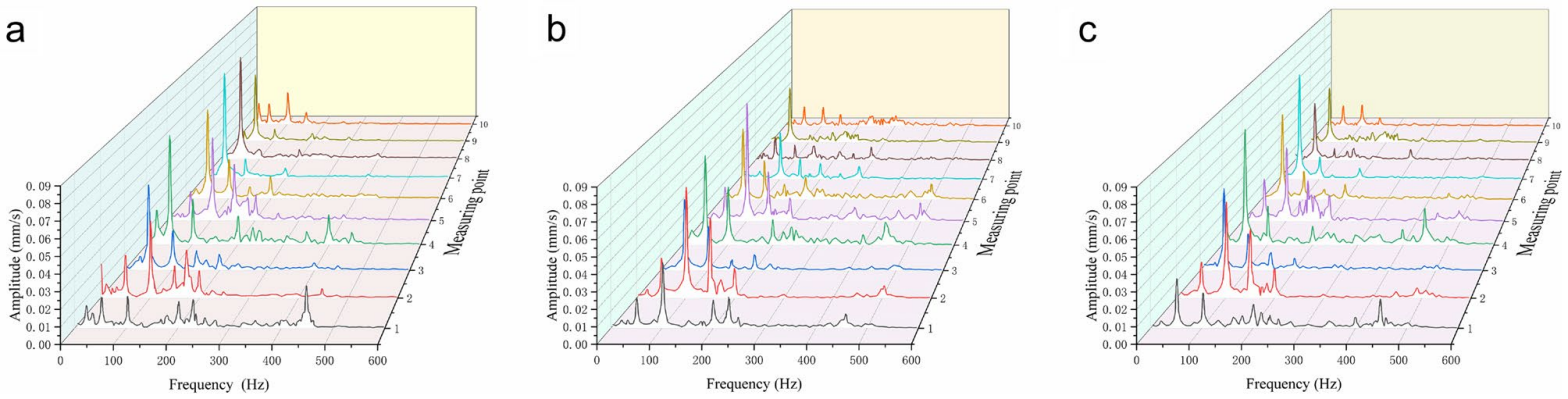
Velocity distribution in frequency-domain of 10 measuring points at (**a**) 0.8Q_d_, (**b**) 1.0Q_d_, (**c**)1.2Q_d_.

Figure 12 shows the frequency-domain distribution of ten different measuring points for three flows of 0.8Q_d_, 1.0Q_d_, and 1.2Q_d_. It could be found that the vibration speed was relatively small at the flow rate of 1.0Q_d_, and the maximum vibration speed was only 0.05 mm/s. At the flow rate of 0.8Q_d_ or 1.2Q_d_, the vibration speed was relatively large, and the maximum vibration speed reached 0.08 mm/s. This showed that the pump ran stably at rated flow with less vibration. However, under the conditions of increasing and decreasing the flow rate, due to the existence of irregular flows such as backflow and leakage flow, the pressure pulsation increased, which in turn caused the fluid exciting force and the vibration speed to increase. At the same time, it could be found that the vibration at the non-drive bearing was larger than that at the drive end bearing. The shaft frequency of each measuring point measured by the test is shown in Table 8. The maximum shaft frequency was 51.8 Hz, and the error between it and the theoretical shaft frequency (49.7 Hz) was about 4.2%. The error of each measuring point might be caused by factors such as motor speed fluctuation, voltage instability, instrument measurement error, and so on.

**Table 8:** Test shaft frequency of each measuring point (unit: Hz).

Point	1	2	3	4	5	6	7	8	9	10
0.8Q_d_ SF	51.8	49.9	50.6	50.6	51.8	50.6	50.6	51.3	51.3	51.3
1.0Q_d_ SF	50.6	50.6	51.2	50.7	51.3	51.3	50.6	50.6	50.7	51.3
1.2Q_d_ SF	50.6	50.6	50.7	51.3	50.6	50.7	51.3	50.6	50.6	50.7

**Figure 13:**
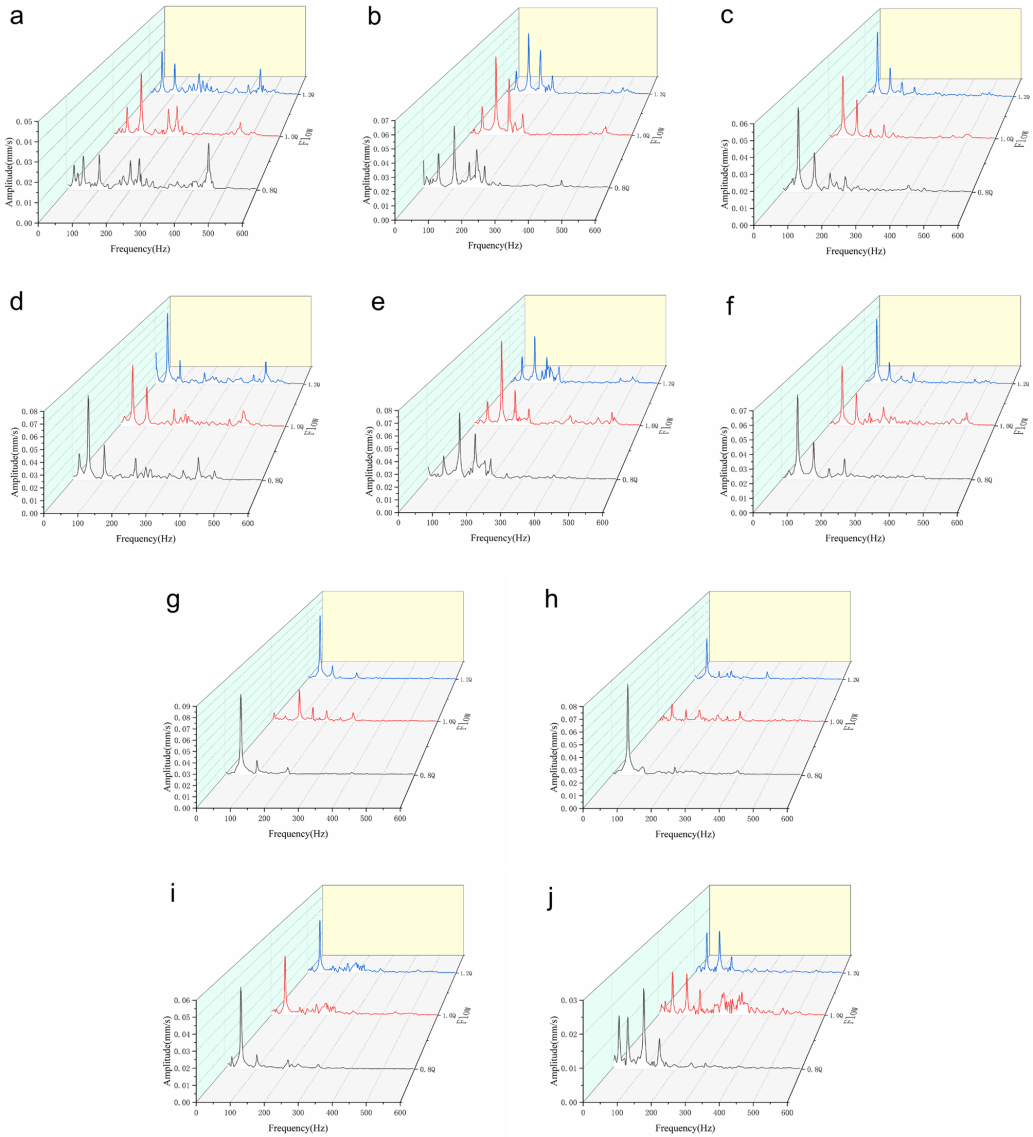
Frequency domain diagrams of 10 measuring points at different flow rates. (**a**) Point 1. (**b**) Point 2. (**c**) Point 3. (**d**) Point 4. (**e**) Point 5. (**f**) Point 6. (**g**) Point 7. (**h**) Point 8. (**i**) Point 9. (**j**) Point 10.

The velocity frequency domain distribution of each vibration measuring point at the flow rate of 0.8Q_d_, 1.0Q_d_, and 1.2Q_d_ is shown in Fig. 13. From Fig. 13, it could be found that the vibration characteristics of the driving bearing and the non-driving bearing in the horizontal direction were the same, the dominant frequency was two times the shaft frequency, and the secondary frequency was three times the shaft frequency. In the axial direction, the dominant frequency of the bearings was the shaft frequency, and the secondary frequency was two times the shaft frequency. The dominant frequency of the non-driven bearing in the vertical direction was the shaft frequency. The above analysis showed that vibration caused by mechanical motion dominates at these five positions. The vertical vibration of the drive bearing end showed different characteristics at different flow rates. At 1.0Q_d_, the dominant frequency was the shaft frequency. At 0.8Q_d_, the amplitude at two times the blade passing frequency increased significantly. At 1.2Q_d_, the dominant frequency became two times the blade passing frequency. This change was mainly because the increase and decrease of the flow rate made the flow in the centrifugal pump unstable, and the vibration caused by the unsteady fluid force was amplified.

The vibration spectrum of the inlet pipe is shown in Fig. 13g. When the centrifugal pump flow rate was 1.2Q_d_ or 0.8Q_d_, the vibration speed reached the maximum amplitude at the shaft frequency, and the maximum amplitude was 0.077 mm/s. At 1.0Q_d_, the amplitude of the vibration velocity was significantly reduced, and the maximum amplitude was only 0.033 mm/s, which appeared at two times the shaft frequency. The vibration spectrum of the outlet pipe is shown in Fig. 13h. The dominant frequency at the three flow rates was the shaft frequency. The highest amplitude was 0.073 mm/s at 0.8Q_d_, 0.042 mm/s at 1.2Q_d_, and only 0.015 mm/s at 1.0Q_d_. The vibration characteristics of the inlet and outlet pipes were greatly affected by the working conditions, and both low flow and high flow conditions would aggravate the vibration^26,30^.

As shown in Fig. 13i, at the vertical direction of the pump casing, the shaft frequency was the dominant frequency. At 0.8Q_d_, the maximum amplitude was higher than in the other two cases, reaching 0.520 mm/s. As shown in Fig. 13j, at the horizontal direction of the pump casing, the maximum amplitudes appeared at two times the shaft frequency and had little difference with the amplitude at the shaft frequency. The vibration spectrum analysis of the vertical and horizontal directions of the pump casing showed that the vibration was aggravated by the low flow condition, while the high flow condition had almost no effect on the vibration.

## Conclusions

In this paper, the modal analysis and vibration test of the seven-stage high-pressure centrifugal pump were carried out, and the simulation and test results were processed and analyzed. The conclusions are as follows:

1. In modal analysis, the centrifugal pump rotor system exhibited vibration modes of swing vibration, torsional vibration, bending vibration, and pitching vibration. The first critical speed of the rotor system was 5270.9 rpm, which was much higher than the rated speed of 2980 rpm. The rotor system would not resonate.
2. The vibration of the multi-stage centrifugal pump at the bearing was periodic, and the period was not only related to the shaft frequency and its frequency multiplication but also related to the blade passing frequency.
3. At rated flow, the vibration peaks of the multistage centrifugal pump appeared at the shaft frequency and its frequency multiplication. Maximum peaks appeared at the shaft frequency (49.7 Hz) and two times the shaft frequency (99.4 Hz).
4. Flow changes had a significant impact on the vibration of the centrifugal pump. The high flow aggravated the vibration of the inlet and outlet pipes, and the low flow aggravated the vibration of the inlet and outlet pipes and pump casing.
5. The vibration of the centrifugal pump rotor system was mainly determined by the SF, two times the SF, three times the SF, the BPF, and two times the BPF. The vibration of the inlet and outlet pipes was mainly determined by the SF and two times the SF. The vibration of the pump casing was mainly determined by the SF, the two times the SF, and the BPF.
6. Mechanical motion is the main factor that causes the pump to vibrate. However, the vibration caused by fluid motion cannot be ignored, especially in the vertical direction of the drive bearing.

The whole study investigates the vibration characteristics of a multi-stage centrifugal pump at different positions. The domain frequency is mainly affected by the shaft frequency and the blade passing frequency. All the results can enrich the existing database. However, the research on the physical mechanism of vibration characteristics is not enough in this paper, and we will strengthen this research in the next step.

## Data Availability

The data that support the findings of this study are available from the corresponding author upon reasonable request.

## List of symbols

Q_d_: Design flow rate (m^3^/h)
n: Rotation speed (rpm)
H: Head (m)
D_in_: Inlet pipe diameter (mm)
D_out_: Outlet pipe diameter (mm)
*Z*: Number of blades
*f*_pf_: Shaft frequency (Hz)
*f*_bpf_: Blade passing frequency (Hz)
SF: Shaft frequency
BPF: Blade passing frequency
